# AEBP1-GLI1 pathway attenuates the FACT complex dependency of bladder cancer cell survival

**DOI:** 10.1016/j.bbrep.2025.102101

**Published:** 2025-06-20

**Authors:** Haruka Kurosu, Norika Yamada, Ritsuko Nakamura, Hideaki Ito, Koji Ohnishi, Akihito Inoko, Miho Riku, Tomoaki Muramatsu, Naoto Sassa, Kenji Kasai

**Affiliations:** a Department of Urology, Aichi Medical University School of Medicine, Japan; b Department of Pathology, Aichi Medical University School of Medicine, Japan

**Keywords:** AEBP1, GLI1, FACT complex, Apoptosis, DNA damage, Bladder cancer

## Abstract

The facilitates chromatin transcription (FACT) complex is composed of SSRP1 and SUPT16H subunits and participates in nucleosomal reorganization; hence, FACT inhibitors are considered promising therapeutics for malignant tumors. Here, we show that adipocyte enhancer binding protein 1 (AEBP1) attenuates the dependency of bladder cancer cell survival on the FACT complex *via* the expression of GLI1, a pivotal transcription factor in Hedgehog signaling. In *AEBP1*-high expressing bladder cancer cell lines, *AEBP1* knockdown inhibited cellular proliferation and induced the marker expression of apoptosis and DNA damage/replication stress. RNA-sequencing revealed that *AEBP1* knockdown suppressed the expression of *SSRP1* and *SUPT16H*; however, the knockdown of both subunits was less effective than *AEBP1* knockdown in inducing apoptosis or DNA damage markers in *AEBP1*-high expressing cells. *AEBP1* knockdown reduced the protein levels of GLI1, and treatment with the GLI-specific inhibitor GANT61 induced markers that were not suppressed by the forced expression of AEBP1. These findings suggest that AEBP1-mediated GLI1 expression reduces the FACT complex dependency of bladder cancer cell survival.

## Highlights

• *AEBP1* depletion induces apoptosis and DNA damage in *AEBP1*-high bladder cancer cells.
• AEBP1 regulates *SSRP1* and *SUPT16H* expression at the transcriptional level.
• *AEBP1* depletion induces apoptosis and DNA damage more than *FACT complex* depletion.
• GLI1 inhibition mimics *AEBP1* depletion on bladder cancer cells.
• AEBP1–GLI1 axis modulates FACT complex–dependent survival mechanisms.

## 1 Introduction

Bladder cancer is clinically divided into non-muscle-invasive (NMIBC) and muscle-invasive (MIBC) bladder cancers, according to the depth of cancer cell invasion. Even with standard treatment, half of the patients with NMIBC recurs, and 10–20 % of the relapsed cases eventually progress to the more aggressive MIBC [1]. MIBC was proposed to be classified into luminal-papillary, luminal non-specified, luminal unstable, stroma-rich, basal/squamous, and neuroendocrine-like molecular subtypes that are strongly linked to gene alterations, including those in *FGFR3*, *KDM6A*, *ELF3*, *PPARG*, *TP53*, *ERCC2,* and *RB1* [1]. However, the therapeutic molecular targets for NMIBC and MIBC are not fully characterized.

Adipocyte enhancer binding protein 1 (AEBP1) is a multifunctional protein that participates in various processes, including adipocyte differentiation [2], cholesterol homeostasis [3], mammary gland development [4,5], and tumorigenesis [[6], [7], [8]]. Gene expression signature analysis revealed that the upregulation of *AEBP1* correlated with the disease progression and poor prognosis of bladder cancer [9]. AEBP1 was initially recognized as a transcriptional repressor; however, it is now known to regulate several pivotal signaling pathways, including those of MAPK, PI3K, PTEN, and NF-kB [7,10]. However, the molecular role of AEBP1 in bladder cancer cells remains unclear.

The facilitates chromatin transcription (FACT) complex consists of SSRP1 and SUPT16H heterodimers and functions as a histone chaperone that regulates nucleosome reorganization [11]. The FACT complex binds to the H2A-H2B histone dimer, leading to either its removal from nucleosomes or relaxation of the nucleosome structure [[12], [13], [14]]. These effects promote the access of Pre-Initiation Complex (PIC) to the promoter region and enhance RNA polymerase II-mediated transcription [15]. The FACT complex is also crucial in DNA damage repair because it promotes the deposition of H2A.X, a histone H2A variant that contributes to chromatin remodeling at sites of DNA damage [16]. Furthermore, it interacts with the minichromosome maintenance (MCM), promoting MCM-mediated DNA unwinding on nucleosome templates and increasing replication speed [17,18]. These evidences support the fundamental role of the FACT complex in transcription, translation, and DNA damage repair. Notably, the FACT complex inhibitor curaxin CBL0137 has been used in clinical trials for several types of malignant tumors, but not for bladder cancer [19].

In this study, using human bladder cancer cell lines, we showed that AEBP1 suppressed the FACT complex dependency of bladder cancer cell survival *via* the expression of GLI1, a pivotal transcription factor in Hedgehog signaling. This indicates a novel therapeutic strategy for *AEBP1*-high expressing bladder cancer using drugs targeting GLI1 but not the FACT complex.

## 2 Materials and methods

### 2.1 Cell lines, and analysis for cell proliferation and cell cycle

Human bladder cancer cell lines were obtained from the RIKEN BRC Cell Bank or DSMZ-German Collection of Microorganisms and Cell Cultures and maintained in RPMI 1640 medium supplemented with 10 % fetal bovine serum (FBS). These cell lines were identified by short tandem repeat (STR) profiles using GenePrint 10 system (Promega). Primary cultures of normal human bladder epithelial cells (HBEC) were obtained from KURABO and maintained in culture medium according to the manufacturer's protocols. For cell proliferation analysis, cells were plated and maintained in above culture medium. After treatment, the cells were peeled off the plate with Accutase (Innovative Cell Technologies), dispersed, and counted using trypan blue exclusion test. For cell cycle analysis, the cells were briefly fixed with 70 % ethanol and treated with 50 μg/mL RNase A and 50 μg/mL propidium iodide (PI). The cell cycle of PI-stained cells was examined using BD LSRFortessa X-20 and analyzed using FlowJo software (Becton Dickinson).

### 2.2 siRNA transfection and lentivirus

siRNA transfection was performed using Lipofectamine RNAiMAX (Thermo Fisher Scientific). siRNAs used in this study were siAEBP1-1,’5-UUGCCUGGAUGGAGAAGAATT-3,’ siAEBP1-4,’5-CAGCUACUACGCACAGAAUTT-3,’ siSSRP1-1, 5′-GACUUAAACUGCUUACAAATT-3,’ siSUPT16H-2, 5′-GUCUAAUGUGUCCUAUAAATT-3,’ siControl, 5′-CGUACGCGGAAUACAACGATT-3’

For creating lentiviruses, pCAG-HIVgp, pCMV-VSV-G, and CSII-CMV-MCS-IRES2-Bsd plasmids were obtained from the RIKEN BRC DNA Bank. Lentiviruses for AEBP1 and its control were prepared using standard techniques. Briefly, human AEBP1 cDNA was cloned into CSII-CMV-MCS-IRES2-Bsd. Lenti-X293T cells (Takara) were transiently transfected with pCAG-HIVgp and pCMV-VSV-G along with either CSII-CMV-MCS-IRES2-Bsd (for Lenti-control) or CSII-CMV-AEBP1-IRES2-Bsd (for Lenti-AEBP1) using Lipofectamine LTX with Plus reagent (Thermo Fisher Scientific). Purified supernatants from the transfected cells were used to transduce Lenti-AEBP1 or Lenti-control into bladder cancer cells. Transduced cancer cells were maintained in a regular culture medium containing blasticidin.

### 2.3 RNA sequence

Briefly, 72 h after siRNA transfection, total RNA was isolated using RNeasy Plus Mini Kit (Qiagen) and used for the construction of a sequencing library using NEBNext Poly(A) mRNA Magnetic Isolation Module and NEBNext Ultra TMII Directional RNA Library Prep Kit (Illumina, Inc.), according to the manufacturer's protocols. Pooled libraries were sequenced using NovaSeq 6000 (Illumina, Inc.) with 150bo x 2 paired-end reads. The quality of the raw paired-end sequence reads was assessed using FastQC (version 0.11.7), and low-quality (<20) bases and adapter sequences were trimmed using Trimmomatic software (version 0.38) with the following parameters: ILLUMINACLIP:path/to/adapter.fa:2:30:10 LEADING:20 TRAILING:20 SLIDINGWINDOW:4:15 MINLEN:36. The trimmed reads were aligned to the human reference genome hg38 using the RNA-seq aligner HISAT2 (version 2.1.0). The abundance of uniquely mapped reads was estimated using featureCounts (version 1.6.3). Raw read counts were normalized using the transcripts per million (TPM) method. Clustering using the Wald method based on the Euclidean distances of the normalized counts was conducted using the stats (version 3.6.1) and gplots (version 3.0.1.1) R packages. Differentially expressed genes (DEGs) were detected with the thresholds of Ilog2(fold change)I > 1 and adjusted p-value <0.05, using the Benjamini and Hochberg method. Raw sequence data were registered in the Gene Expression Omnibus (GEO) database (NCBI) under the accession number GSE288105.

### 2.4 Immunoblot analysis and antibodies

Protein samples were extracted from the cells using sample buffer containing 50 mM Tris-HCl pH 6.8, 2 % SDS, 10 % glycerol, complete mini protease inhibitor (Roche) and dithiothreitol (DTT). The samples were separated by SDS-PAGE and transferred to polyvinylidene difluoride membranes (Millipore). The membranes were blocked with either 5 % skim milk or BSA in Tris-buffered saline containing 0.02 % Tween-20 and incubated with primary antibodies. After washing, the membranes were incubated with HRP-labelled secondary antibodies, followed by detection with ECL reagent (Cytiva). Luminescent images were analyzed using Amersham Imager 600 (GE Healthcare Life Science). The signal intensity was measured using ImageJ software. Antibodies used in this study are listed in Supplementary Table S1.

## 3 Results

### 3.1 AEBP1 expression in bladder cancer cell lines

Publicly available gene chip data predicted that the expression of *AEBP1* in bladder cancer tissues is lower than that of normal bladder tissues, although the difference was not statistically significant (Fig. 1A). However, high *AEBP1* expression negatively affected the overall survival of patients with bladder cancer (*P* = 0.002) (Fig. 1B), indicating a biological difference between high and low *AEBP1* expression in bladder cancers. Immunoblot analyses revealed that the expression of AEBP1 in human bladder cancer cell lines 5637 and KU1919 was higher than that of other seven bladder cancer cell lines (253J, J82, RT112, JMSU1, VMCUB1, BOY12E, and SW1710) and the primary culture of normal human bladder epithelial cells HBEC (Fig. 1C). So, we used 5637, KU1919 (*AEBP1*-high), and JMSU1 (*AEBP1*-low) bladder cancer cell lines to examine the role of AEBP1. The proliferation of 5637 and KU1919 cells was slower than that of JMSU1 cells (Fig. 1D). We next established JMSU1 cells infected with either AEBP1-expressing lentivirus (Lenti-AEBP1) or its control (Lenti-control) (Supplementary Fig. S1A). Using these cells, we found that AEBP1 slightly but not statistically significantly suppressed the cellular proliferation of JMSU1 cells (Supplementary Fig. S1B), even with no apparent change of cell cycle (Supplementary Fig. S1C; Supplementary Table S2 for raw data from three independent experiments). These results indicated that a poorer prognosis of patients with *AEBP1*-high bladder cancer was not directly associated with the proliferation rate of cancer cells. Intriguingly, we found that siRNA-mediated knockdown of *AEBP1* significantly reduced the viability of 5637 and KU1919 cells, indicating the *AEBP1*-dependent survival of these *AEBP1*-high cancer cells (Fig. 1E and F).

**Fig. 1:**
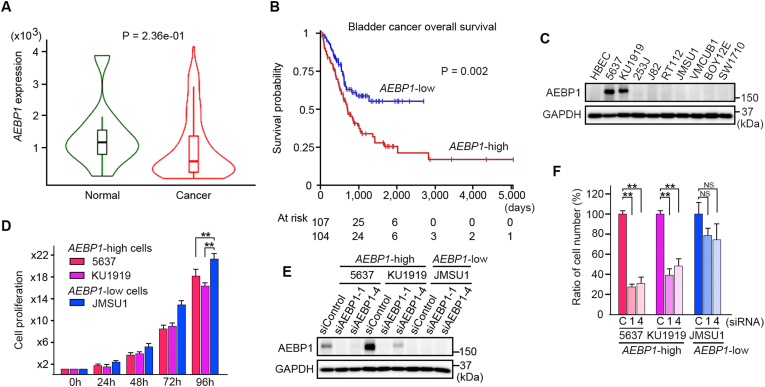
*AEBP1* expression in bladder cancer cell lines. A, Gene chip analysis for *AEBP1* expression in normal and cancer bladders using TNMplot software (https://tnmplot.com/analysis/). B, Kaplan-Meier analysis of overall survival of patients with bladder cancer using UCSCxena software (https://xena.ucsc.edu/). High expression of *AEBP1* negatively impacts overall survival. C, AEBP1 expression in normal human bladder epithelial cells (HBEC) and bladder cancer cell lines. D, Cell proliferation of *AEBP1*-high (5637 and KU1919) and *AEBP1*-low (JMSU1) cells. Data are shown as mean + SD from six biological replicates. *P*‐values obtained by Student's t‐tests. *P* < 0.001 was set as statistically significant. Double asterisk, *P* < 0.001. E, Analyses of *AEBP1* knockdown. 72 h after transfection with siRNAs for *AEBP1* (siAEBP1-1 and siAEBP1-4) or their control (siControl), cells were harvested for the immunoblot analyses. F, Ratios of cell numbers 72 h after transfection of siControl (C), siAEBP1-1 (1), or siAEBP1-4 (4). Data are shown as mean + SD from four or three biological replicates. *P*‐values obtained by Student's t‐tests. *P* < 0.001 was considered as statistically significant. Double asterisk, *P* < 0.001. NS, not significant.

### 3.2 AEBP1-dependent cell cycle progression in AEBP1-high cancer cells

Next, we performed cell cycle analyses of siRNA-transfected cells and measured the cell cycle fractions of 5637, KU1919, and JMSU1 cells 72 h after siRNA transfection. *AEBP1* knockdown in 5637 and KU1919 cells—but not JMSU1 cells—increased the fractions of subG1-phase and S-phase cells (Fig. 2A; Supplementary Table S3 for raw data from three independent experiments). Immunoblot analyses revealed that *AEBP1* knockdown in 5637 and KU1919 cells—but not in JMSU1 cells—induced the cleaved forms of PARP (cPARP) and Caspase3 (cCaspase3), which are markers of apoptotic cell death, as well as increased levels of phosphorylated histone H2A (γ-H2AX), a marker of DNA damage and replication stress (Fig. 2B). DNA damage response (DDR) is required for genomic integrity and steady S-phase progression of either normal or cancer cells, and impaired DDR induces excessive DNA damage and replication stress, leading to cell cycle alteration or cell death. Among factors involved in DDR, Ataxia Telangiectasia and Rad3-related (ATR) and its downstream Checkpoint kinase 1 (Chk1) are central players of DDR, and ATR-Chk1 activity reportedly mitigates replication stress and maintains S-phase progression [20]. Consistent with the cell cycle alteration and marker induction shown in Fig. 2A and B, *AEBP1* knockdown in 5637 and KU1919 cells—but not JMSU1 cells—suppressed the phosphorylation of ATR along with reduction of the upper sized band of Chk1, which indicated the reduction of phosphorylated Chk1 (Fig. 2C). These results revealed that the survival of 5637 and KU1919 cells depended on AEBP1, probably *via* maintenance of their DDR activity.

**Fig. 2:**
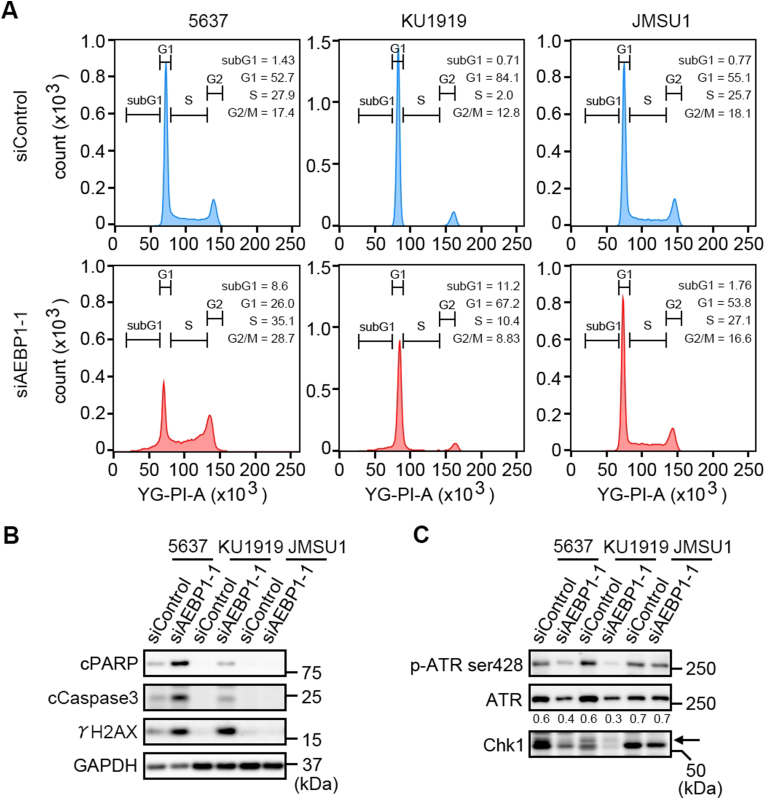
AEBP1-dependent cell cycle progression in *AEBP1*-high cancer cells. A, Cell cycle analyses of siRNA-transfected cells using flow cytometry. Cells were analyzed 72 h after siRNA transfection. The experiments were conducted three times and representative data are shown. The raw data regarding the fraction of each cell cycle phase from three experiments is listed in Supplementary Table S3. B, Induction of the cleaved form of PARP (cPARP), Caspase3 (cCaspase3), and the phosphorylated form of histone H2A (γ-H2AX) by *AEBP1* knockdown. Cells were harvested for the immunoblot analyses 72 h after siRNA transfection. C, Expression of phosphorylated ATR (*p*-ATR ser428), ATR and Chk1. *AEBP1* knockdown reduced phosphorylated ATR in 5637 and KU1919 cells. Note that the upper sized band of Chk1 was reduced in *AEBP1*-knockdown 5637 and KU1919 cells. Ratios of *p*-ATR against ATR were semi-quantified using ImageJ software.

### 3.3 FACT complex independency of AEBP1-high cancer cells

To further explore the molecular effects of *AEBP1* knockdown, we compared the gene expression between *AEBP1*-knockdown 5637 and control cells using RNA-seq (Fig. 3A; raw data were registered in the GEO database (NCBI) under the accession number GSE288105). Among several DEGs, we found that those related to chromatin modification (*SMARCA2*, *SMARCC2*, *BRD7*, *HMGB2*, *RNF20*, *BUB1B*, *SSRP1* and *SUPT16H*) were decreased by *AEBP1* knockdown (Fig. 3A). In this study, we focused on the decreased expression of *SSRP1* and *SUPT16H*. SSRP1 and SUPT16H are subunits of the FACT complex, a histone chaperone essential for the dynamic nature of the chromatin structure during the cell cycle [11]. Considering their reported fundamental roles in cell cycle progression, SSRP1 and SUPT16H proteins were detected in HBEC and bladder cancer cell lines with low or undetectable levels of AEBP1 (Fig. 3B). Hence, the expression of *SSRP1* and *SUPT16H* was not solely regulated by AEBP1.

**Fig. 3:**
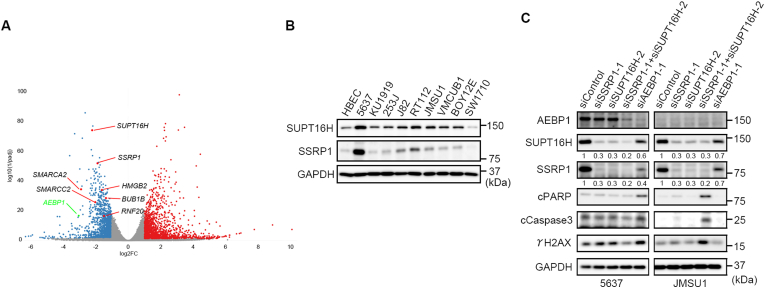
FACT complex independency of *AEBP1*-high cancer cells. A, Volcano plot of RNA-seq data. 72 h after transfection with either siControl or siAEBP1-1, 5637 cells were harvested for RNA extraction followed by RNA-seq analyses. Blue and red dots show down- and upregulated genes by *AEBP1* knockdown, respectively. RNA-seq data was registered in the GEO database (NCBI) under the accession number GSE288105. B, Expression of SSRP1 and SUPT16H proteins in normal human bladder epithelial cells (HBEC) and bladder cancer cell lines. C, Induction of the cleaved form of PARP (cPARP), Caspase3 (cCaspase3), and the phosphorylated form of histone H2A (γ-H2AX). Cells were harvested for the immunoblot analyses 72 h after siRNA transfection. Double knockdown of *SSRP1* and *SUPT16H* enhances the induction of cPARP, cCaspase3, and γ-H2AX in JMSU1 but not 5637 cells. In 5637 cells, *AEBP1* knockdown induces these markers more than *SSRP1* and *SUPT16H* double knockdown. Note that consistent with RNA-seq data, *AEBP1* knockdown reduced the protein levels of SSRP1 and SUPT16H in 5637 cells, but not in JMSU1 cells. The signal intensity of SUPT16H and SSRP1 was semi-quantified using ImageJ software.

The FACT complex is essential for DNA damage repair and the resolution of DNA replication stress. Therefore, we examined the effect of the FACT complex knockdown and compared it with *AEBP1* knockdown in 5637 and JMSU1 cells. The FACT complex is stable only when SSRP1 and SUPT16H subunits are present and form a complex [21,22]. Consistently, we found that the single knockdown of either subunit reduced the protein levels of the other subunit (Fig. 3C). However, in JMSU1 cells, the double knockdown of *SSRP1* and *SUPT16H* effectively induced cPARP, cCaspase3, and γ-H2AX more than the single knockdown of either subunit, indicating that a single knockdown did not completely diminish the expression of both proteins. Under these experimental conditions, double knockdown of *SSRP1* and *SUPT16H* in 5637 cells induced lower levels of these apoptotic and DNA damage markers compared with *AEBP1* knockdown (Fig. 3C). Therefore, we hypothesized that AEBP1 maintained the expression of the FACT complex and participated in the survival of 5637 cells by a mechanism other than the FACT complex.

### 3.4 GLI1 defines the FACT complex independency in AEBP1-high cancer cells

We previously reported that GLI1, a transcription factor of the Hedgehog signaling, enhances the promoter activity of *AEBP1* in breast cancer cells [23]. Accordingly, the TCGA database of bladder cancer indicated a weak correlation between *AEBP1* expression and *GLI1* (rho = 0.5366, p = 4.004e-33) and a strong correlation with *GLI2* (rho = 0.7954, p = 3.731e-94) (Fig. 4A). Immunoblot analyses revealed that 5637 and KU1919 cells highly expressed GLI1 and GLI2 proteins (Fig. 4B). However, in our preliminary experiments using human *AEBP1* promoter-driven luciferase reporter plasmid, the forced expression of either GLI1 or GLI2 failed to upregulate *AEBP1* promoter activity in bladder cancer cells (data not shown). Instead, we found that *AEBP1* knockdown in 5637 and KU1919 cells reduced the protein levels of GLI1, but not GLI2 (Fig. 4C).

**Fig. 4:**
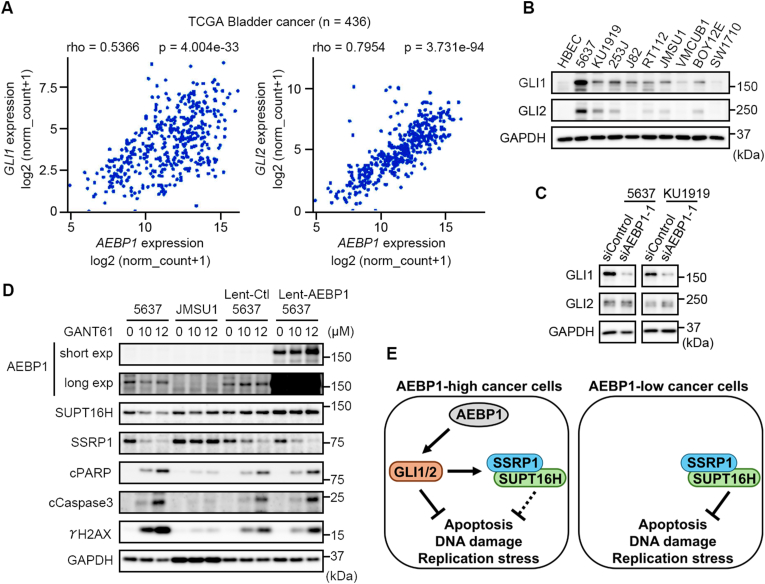
GLI1 defines the FACT complex independency in *AEBP1*-high cancer cells. A, Correlated expression of *GLI1* and *GLI2* with *AEBP1* in bladder cancer. TCGA data (n = 436) was analyzed using USCSxena software (https://xena.ucsc.edu/). B, Expression of GLI1 and GLI2 proteins in normal human bladder epithelial cells (HBEC) and bladder cancer cell lines. C, *AEBP1* knockdown reduces GLI1 protein in 5637 and KU1919 cells. D, GANT61-induced cPARP, cCaspase3, and γ-H2AX were not suppressed by *AEBP1* overexpression. Cells were treated with GANT61 (10 and 12 μM) or its vehicle DMSO (0 μM) for 72 h. For the overexpression experiments, 5637 cells were transduced with either AEBP1 expressing lentivirus (Lenti-AEBP1) or its empty control lentivirus (Lenti-control) and maintained under blasticidin. Immunoblot analyses detected the expression of endogenous and virally transduced AEBP1 *via* short or long exposure. E, Scheme of AEBP1 role in cancer cell survival. In *AEBP1*-high cancer cells, GLI1 bypasses the FACT complex to maintain the cell survival, resulting in limited FACT complex dependency of cancer cell survival.

GLI1 participates in the proliferation and survival of many cancer cell types [24,25]. Therefore, we assumed GLI1-dependent cell survival in *AEBP1*-high cancer cells. To clarify this, we employed GANT61, inhibitor for GLI1 and GLI2, and found that its application to 5637 cells—but not to JMSU1 cells—induced cPARP, cCaspase3, and γ-H2AX in a dose-dependent manner (Fig. 4D). The protein levels of AEBP1 and SUPT16H in 5637 cells were not significantly reduced by GANT61. However, GANT61 reduced the protein levels of SSRP1 in 5637 cells but not in JMSU1 cells. Because GANT61 suppresses the function of GLI1 and GLI2, this result did not determine which GLI was involved. However, with the evidence that *AEBP1* knockdown reduced GLI1 but not GLI2 (Fig. 4C), we speculated that SSRP1 in *AEBP1*-high cancer cells was mainly maintained by GLI1 (Fig. 4D). To clarify the AEBP1-GLI1-SSRP1 relationship, we established 5637 cells infected with either AEBP1-expressing lentivirus (Lenti-AEBP1) or its control (Lenti-control); these 5637 cells were treated with GANT61 and immunoblot analyses were performed. We found that the forced expression of AEBP1 did not suppress GANT61-mediated induction of cPARP, cCaspase3, and γ-H2AX, indicating that AEBP1-dependent survival of 5637 cells was mainly mediated by GLI1 (Fig. 4D). Importantly, GANT61 treatment of Lenti-AEBP1 5637 cells reduced the levels of SSRP1, but not those of SUPT16H, supporting again that SSRP1 was under the control of GLI1. Furthermore, considering that *SSRP1* and *SUPT16H* double knockdown in 5637 cells was less effective than those in JMSU1 cells to induce cPARP, cCaspase3, and γ-H2AX (Fig. 3C), and also that GANT61 treatment reduced phosphorylated ATR (and Chk1), even in Lenti-AEBP1 5637 cells (Supplementary Fig. S2), we concluded that AEBP1-mediated GLI1 expression attenuated the FACT complex-dependency of bladder cancer cell survival, probably *via* GLI1-mediated maintenance of the DDR activity (Fig. 4E).

## 4 Discussion

The FACT complex participates in several cell cycle regulation processes [11]. For instance, *SSRP1*-depletion in chicken DT40 cells delays S-phase progression and reduces the DNA replication fork rate without activating the ATR-Chk1 checkpoint response [18]. Under hydroxyurea treatment to induce DNA replication stress, *SSRP1*-knockdown MCF10A cells lost the ATR-Chk1 checkpoint response and showed increased susceptibility to replication-induced DNA damage [22]. In the present study, we consistently showed that double knockdown of *SSRP1* and *SUPT16H* induced cPARP, cCaspase3, and γ-H2AX in JMSU1 cells. However, in 5637 cells, *AEBP1*-knockdown more efficiently induced these apoptotic and DNA damage markers than the double knockdown, and GANT61-mediated induction of these markers was not rescued by the forced expression of AEBP1. Considering that *AEBP1* knockdown reduced GLI1 levels in *AEBP1*-high cells, our findings suggest that GLI1 is downstream of AEBP1 and relieves the effect of FACT complex depletion. GLI1 reportedly upregulates the transcription of *FANCD2* [26], which is involved in the stabilization of DNA replication forks and the repair of collapsed forks. GLI1 also upregulates *Bid* transcription and enhances the phosphorylation of Chk1 in the ATR-Chk1 pathway [27]. Combined with other studies showing GLI1 involvement in the activation of DNA damage repair and the reduction of replication stress [28,29], our findings suggest that GLI1 partly restores the FACT complex knockdown-induced DNA damage and replication stress through its target genes. This possibility was also supported by our results that *AEBP1* knockdown reduced the phosphorylation of ATR (and Chl1), which was also suppressed by GANT61 in AEBP1-overexpressing 5637 cells (Fig. 2C and Supplementary Fig. S2).

Previous reports from our group and others have shown that the expression and function of GLI1 and GLI2 is regulated through mechanisms such as protein degradation [30], subcellular localization [31], and binding with other transcriptional regulators [23,32]. In this study, we showed that AEBP1 participates in maintaining the protein levels of GLI1 in bladder cancer cells. Hence, consistent with previous reports [4,6,8,33], we think that the downstream pathways of AEBP1 such as NF-kB pathway is involved in the regulation of GLI1.

The therapeutic potential of the FACT complex inhibitor curaxin CBL0137 has been proposed for several types of malignant tumors [19]. However, to the best of our knowledge, no clinical trials of CBL0137 in bladder cancer have been reported. Considering our findings, we believe that targeting GLI1, not the FACT complex, would be therapeutically useful for patients with *AEBP1*-high bladder cancer.

## CRediT authorship contribution statement

**Haruka Kurosu:** Writing – review & editing, Writing – original draft, Methodology, Investigation, Data curation. **Norika Yamada:** Validation, Investigation. **Ritsuko Nakamura:** Writing – review & editing, Investigation. **Hideaki Ito:** Writing – review & editing, Methodology. **Koji Ohnishi:** Writing – review & editing. **Akihito Inoko:** Writing – review & editing, Data curation. **Miho Riku:** Writing – review & editing, Methodology. **Tomoaki Muramatsu:** Visualization, Methodology. **Naoto Sassa:** Writing – review & editing, Project administration, Funding acquisition. **Kenji Kasai:** Writing – review & editing, Writing – original draft, Supervision, Resources, Project administration, Methodology, Funding acquisition, Data curation, Conceptualization.

## Funding

This work was partially supported by JSPS KAKENHI (Grant Number 23K06505 (KK)).

## Declaration of competing interest

The authors declare that they have no competing financial interests or personal relationships that could have influenced the work reported in this study.

## Figures and Tables

**figs1:**
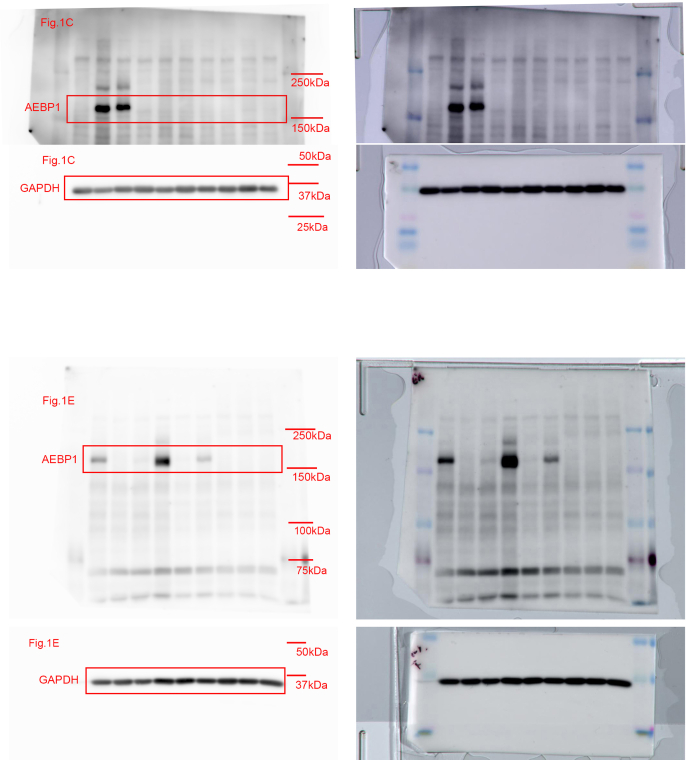


**figs2:**
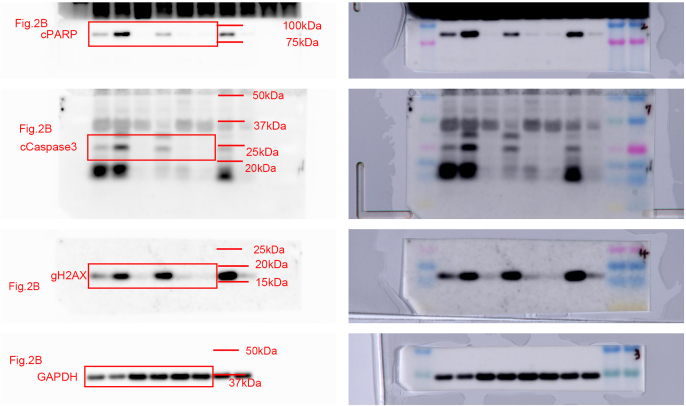


**figs3:**
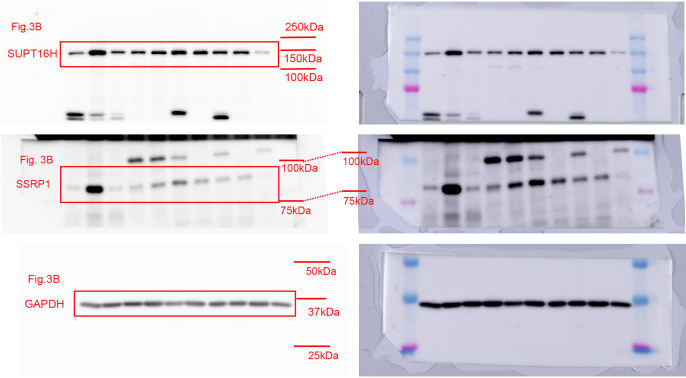


**figs4:**
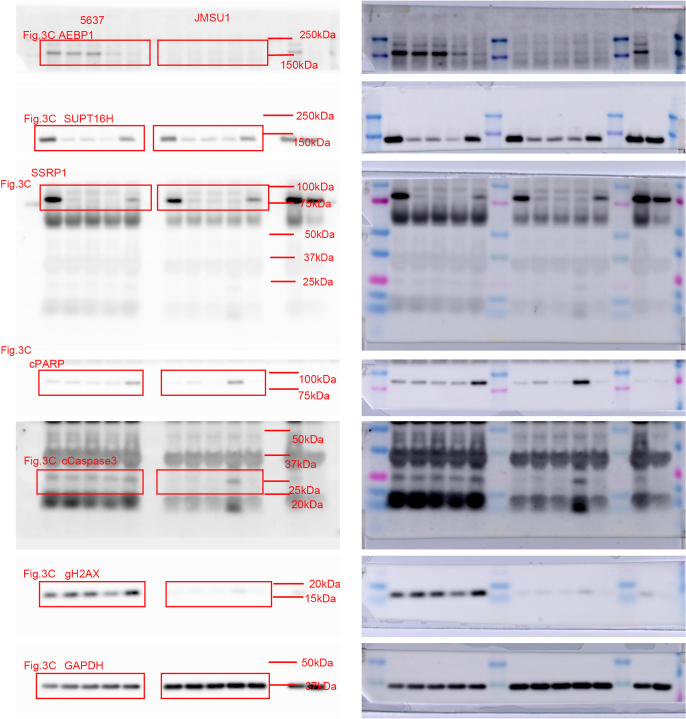


**figs5:**
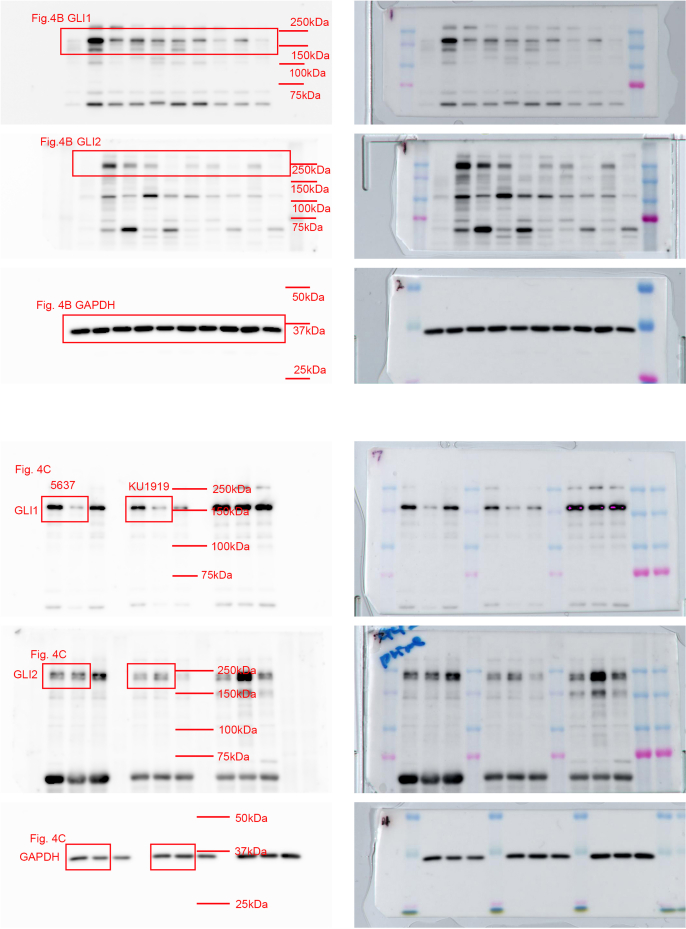


**figs6:**
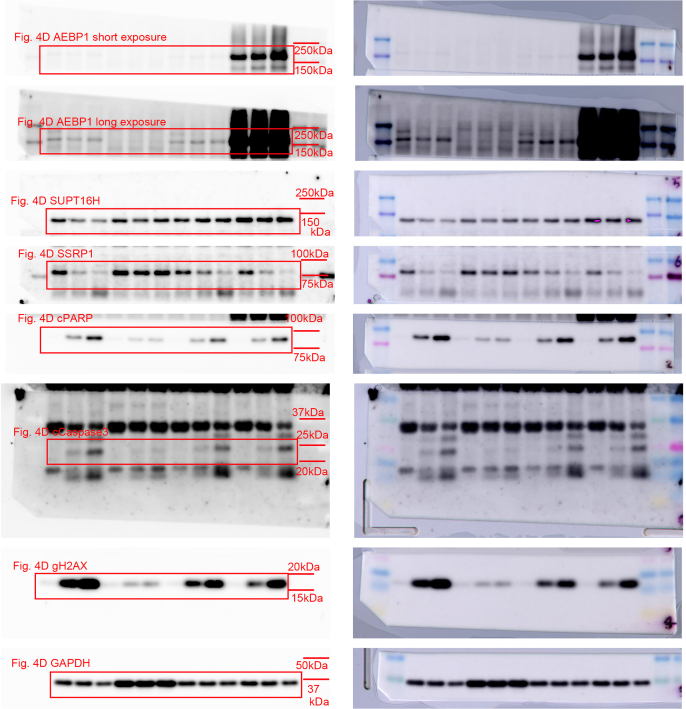


**figs7:**
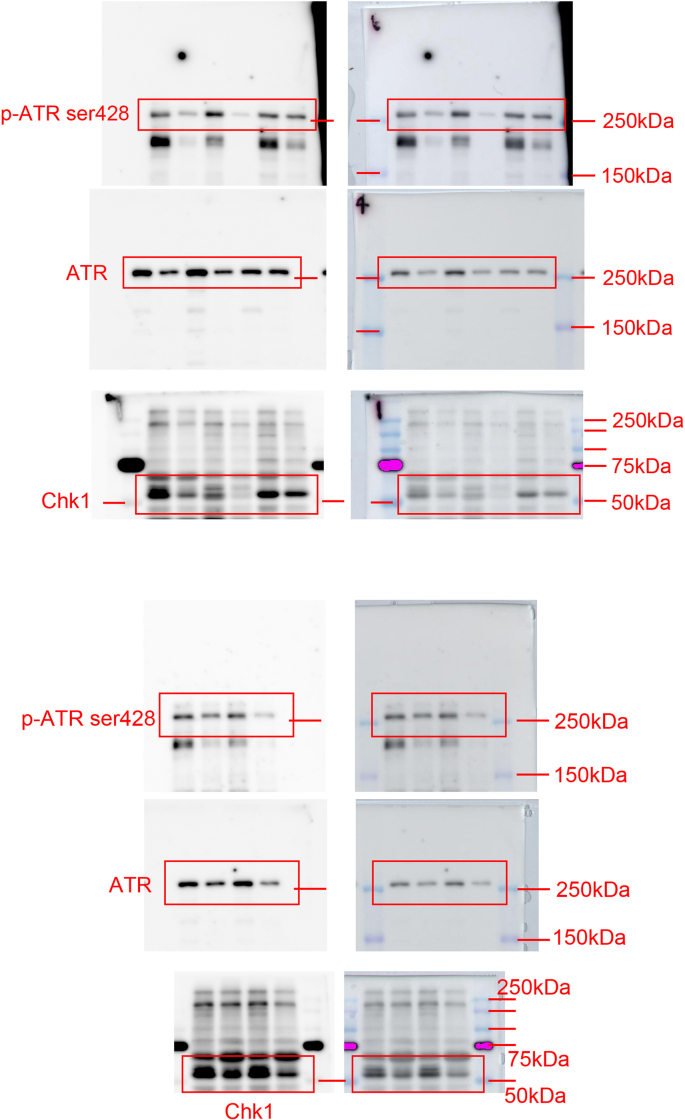


**figs8:**
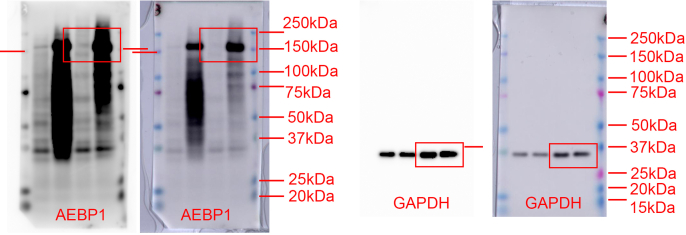


## Data Availability

Data will be made available on request.
