# Supplementary material for: AEBP1-GLI1 pathway attenuates the FACT complex dependency of bladder cancer cell survival
Source: Biochem Biophys Rep. 2025 Jun 20;43:102101. doi: 10.1016/j.bbrep.2025.102101 (PMC12221834; doi:10.1016/j.bbrep.2025.102101)
Supplement: Multimedia component 1 [file mmc1.docx]

**Supplementary Figure_S1.**

**Supplementary Fig. S1. The forced expression of AEBP1 in JMSU1 cells.**

A, Immunoblot analyses of JMSU1 cells lentivirally transduced with either AEBP1 (Lenti-AEBP1) or its control (Lenti-Ctl). B, Cell proliferation assay of lentivirally transduced JMSU1 cells. Note that the forced expression of AEBP1 slightly but not statistically significanly suppresses the cellular proliferation of JMSU1 cells. Data are shown as mean + SD from four biological replicates. *P*‐values obtained by Student’s t‐tests. *P* < 0.001 was considered as statistically significant. NS, not significant. C, Cell cycle analyses of lentivirally transduced JMSU1 cells. The experiments were conducted three times and representative data are shown. The raw data regarding the fraction of each cell cycle phase from three experiments is listed in Supplementary Table S2.
