# Supplementary material for: AEBP1-GLI1 pathway attenuates the FACT complex dependency of bladder cancer cell survival
Source: Biochem Biophys Rep. 2025 Jun 20;43:102101. doi: 10.1016/j.bbrep.2025.102101 (PMC12221834; doi:10.1016/j.bbrep.2025.102101)
Supplement: Multimedia component 2 [file mmc2.docx]

**Supplementary Figure_S2.**

**Supplementary Fig. S2. GANT61 suppressed the phosphorylation of ATR and Chk1.** Immunoblot analyses of 5637 cells lentivirally transduced with either AEBP1 (Lenti-AEBP1) or its control (Lenti-Ctl). The cells were treated with GANT61 (12 μM) or its vehicle DMSO (0 μM) for 72 hours. The ratios of phosphorylated ATR against ATR were semi-quantified using ImageJ software.
