# Supplementary material for: AEBP1-GLI1 pathway attenuates the FACT complex dependency of bladder cancer cell survival
Source: Biochem Biophys Rep. 2025 Jun 20;43:102101. doi: 10.1016/j.bbrep.2025.102101 (PMC12221834; doi:10.1016/j.bbrep.2025.102101)
Supplement: Multimedia component 3 [file mmc3.docx]

**Supplementary Table S1.**

**Primary antibody Catalog number Company**


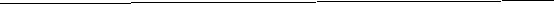


AEBP1 PA5-109366 Invitrogen

SUPT16H sc-165987 Santa Cruz Biotechnology

SSRP1 sc-74536 Santa Cruz Biotechnology

GLI1 sc-515751 Santa Cruz Biotechnology

GLI2 sc-28674 Santa Cruz Biotechnology

GAPDH sc-32233 Santa Cruz Biotechnology

cleaved caspase3 Asp214 5A1E #9664 Cell Signaling Technology

cleaved PARP D64E10 #5625 Cell Signaling Technology

phosphorylated ATR ser428 #2853 Cell Signaling Technology

ATR #2790 Cell Signaling Technology

Chk1 #2345 Cell Signaling Technology

phospho-Histone H2A.X Ser139 JBW301 #05-636 Merck

**Secondary antibody Catalog number Company**


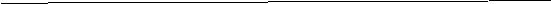


goat anti-mouse immunoglobulins-HRP P0447 DAKO

goat anti-rabbit immunoglobulins-HRP P0448 DAKO

**Supplementary Table S1. List of antibodies used in this study**

Primary and secondary antibodies used for the immunoblot analyses are listed.
