# Supplementary material for: AEBP1-GLI1 pathway attenuates the FACT complex dependency of bladder cancer cell survival
Source: Biochem Biophys Rep. 2025 Jun 20;43:102101. doi: 10.1016/j.bbrep.2025.102101 (PMC12221834; doi:10.1016/j.bbrep.2025.102101)
Supplement: Multimedia component 4 [file mmc4.docx]

**Supplementary Table S2.**

|  | Lenti-Ctl | | | | Lenti-AEBP1 | | | |
| --- | --- | --- | --- | --- | --- | --- | --- | --- |
|  | subG1(%) | G1 (%) | S (%) | G2/M(%) | subG1(%) | G1 (%) | S (%) | G2/M(%) |
| JMSU1 | 0.18 | 47.7 | 30.3 | 21.5 | 0.20 | 46.4 | 30.7 | 22.2 |
|  | 0.24 | 47.3 | 30.2 | 21.9 | 0.12 | 46.9 | 30.2 | 22.4 |
|  | 0.20 | 46.0 | 31.9 | 21.6 | 0.29 | 47.6 | 29.5 | 22.4 |

**Supplementary Table S2. Cell cycle analyses of lentivirus-transfected JMSU1 cells.**

Bladder cancer cell line JMSU1 was lentivirally transduced with either AEBP1 (Lenti-AEBP1) or its control (Lenti-Ctl). Transduced cancer cells were maintained in a regular culture medium containing blasticidin. For cell cycle analysis, the cells were briefly fixed with 70% ethanol and treated with 50 μg/mL RNase A and 50 μg/mL propidium iodide (PI). The cell cycle of PI-stained cells was examined using BD LSRFortessa X-20 and analyzed using FlowJo software (Becton Dickinson).
