# Supplementary material for: AEBP1-GLI1 pathway attenuates the FACT complex dependency of bladder cancer cell survival
Source: Biochem Biophys Rep. 2025 Jun 20;43:102101. doi: 10.1016/j.bbrep.2025.102101 (PMC12221834; doi:10.1016/j.bbrep.2025.102101)
Supplement: Multimedia component 5 [file mmc5.docx]

**Supplementary Table S3.**

|  | siControl transfection | | | | siAEBP1-1 transfection | | | |
| --- | --- | --- | --- | --- | --- | --- | --- | --- |
|  | subG1(%) | G1 (%) | S (%) | G2/M(%) | subG1(%) | G1 (%) | S (%) | G2/M(%) |
| 5637 | 1.43 | 52.7 | 27.9 | 17.4 | 8.6 | 26.0 | 35.1 | 28.7 |
|  | 1.91 | 52.2 | 28.9 | 16.3 | 8.36 | 28.9 | 33.4 | 27.4 |
|  | 1.75 | 52.6 | 28.4 | 16.8 | 8.84 | 28.1 | 35.6 | 25.8 |
| KU1919 | 0.71 | 84.1 | 2.04 | 12.8 | 11.2 | 67.2 | 10.4 | 8.83 |
|  | 0.84 | 84.8 | 1.65 | 12.2 | 11.0 | 70.1 | 9.81 | 7.67 |
|  | 0.90 | 84.3 | 1.91 | 12.5 | 10.9 | 71.0 | 9.05 | 8.81 |
| JMSU1 | 0.77 | 55.1 | 25.7 | 18.1 | 1.76 | 53.8 | 27.1 | 16.6 |
|  | 0.71 | 54.4 | 26.7 | 17.7 | 1.64 | 53.6 | 26.5 | 17.7 |
|  | 0.51 | 55.4 | 26.3 | 17.3 | 1.64 | 53.6 | 26.9 | 17.4 |

**Supplementary Table S3. Cell cycle analyses of siRNA-treated cancer cells.**

Bladder cancer cell lines, 5637, KU1919 and JMSU1, were transfected with either siControl or siAEBP1-1. 72 hours after transfection, the fractions of each cell cycle were measured. For cell cycle analysis, the cells were briefly fixed with 70% ethanol and treated with 50 μg/mL RNase A and 50 μg/mL propidium iodide (PI). The cell cycle of PI-stained cells was examined using BD LSRFortessa X-20 and analyzed using FlowJo software (Becton Dickinson).
