# Supplementary material for: AEBP1-GLI1 pathway attenuates the FACT complex dependency of bladder cancer cell survival
Source: Biochem Biophys Rep. 2025 Jun 20;43:102101. doi: 10.1016/j.bbrep.2025.102101 (PMC12221834; doi:10.1016/j.bbrep.2025.102101)
Supplement: Multimedia component 6 [file mmc6.docx]

June 10, 2025

Elsevier Editorial Office

*Biochemistry and Biophysics Reports*

**RE: Manuscript Number: BBREP-D-25-00931**

Title: AEBP1-GLI1 pathway attenuates the FACT complex dependency of bladder cancer cell survival

Please find attached files showing uncropped Western blot images. These files are associated with our revised submission to *BBR*, titled “AEBP1-GLI1 pathway attenuates the FACT complex dependency of bladder cancer cell survival”. These files include the images from the experiments conducted for the revised manuscript in addition to those for the original submission.

These files include gray images with red marks (protein name and protein size marker) and marker-combined color images obtained using luminescent image analyzer.

I would appreciate it if you find these files would be suitable for editorial process.

Thank you for your consideration. I look forward to hearing from you.

Sincerely,

Kenji Kasai, MD, PhD.

Professor, Department of Pathology

Aichi Medical University School of Medicine

1-1 Yazakokarimata, Nagakute, 480-1195, Aichi Japan

E-mail address: kkasai@aichi-med-u.ac.jp
